# Smoking Prevalence among Physicians: A Systematic Review and Meta-Analysis

**DOI:** 10.3390/ijerph182413328

**Published:** 2021-12-17

**Authors:** Anaïs Besson, Alice Tarpin, Valentin Flaudias, Georges Brousse, Catherine Laporte, Amanda Benson, Valentin Navel, Jean-Baptiste Bouillon-Minois, Frédéric Dutheil

**Affiliations:** 1 Family Medicine, University Hospital of Clermont-Ferrand, Université Clermont Auvergne, F-63000 Clermont-Ferrand, France; bessona.ab@gmail.com (A.B.); alice.tarpin@gmail.com (A.T.); 2 Univ Angers, Laboratoire de psychologie des Pays de la Loire, Université de Nantes, LPPL, EA 4638, F-44000 Nantes, France; valentin.flaudias@univ-nantes.fr; 3 Clermont Auvergne INP, CHU Clermont-Ferrand, CNRS, Institut Pascal, Université Clermont Auvergne, F-63000 Clermont–Ferrand, France; gbrousse@chu-clermontferrand.fr (G.B.); catherinelaporte63@gmail.com (C.L.); 4 Sport Innovation Research Group, Department of Health and Biostatistics, Swinburne University of Technology, Melbourne, VIC 3122, Australia; abenson@swin.edu.au; 5 CNRS, INSERM, GReD, Translational Approach to Epithelial Injury and Repair, CHU Clermont-Ferrand, Ophthalmology, Université Clermont Auvergne, F-63000 Clermont-Ferrand, France; valentin.navel@hotmail.fr; 6 CNRS, LaPSCo, Physiological and Psychosocial Stress, University Hospital of Clermont-Ferrand, Emergency Medicine, Université Clermont Auvergne, F-63000 Clermont-Ferrand, France; 7 CNRS, LaPSCo, Physiological and Psychosocial Stress, University Hospital of Clermont-Ferrand, Occupational and Environmental Medicine, Université Clermont Auvergne, WittyFit, F-63000 Clermont-Ferrand, France; fdutheil@chu-clermontferrand.fr

**Keywords:** tobacco, smoking, physician, doctor, prevalence

## Abstract

Background: Smoking is a major public health problem. Although physicians have a key role in the fight against smoking, some of them are still smoking. Thus, we aimed to conduct a systematic review and meta-analysis on the prevalence of smoking among physicians. Methods: PubMed, Cochrane, and Embase databases were searched. The prevalence of smoking among physicians was estimated and stratified, where possible, by specialties, continents, and periods of time. Then, meta-regressions were performed regarding putative influencing factors such as age and sex. Results: Among 246 studies and 497,081 physicians, the smoking prevalence among physicians was 21% (95CI 20 to 23%). Prevalence of smoking was 25% in medical students, 24% in family practitioners, 18% in surgical specialties, 17% in psychiatrists, 16% in medical specialties, 11% in anesthesiologists, 9% in radiologists, and 8% in pediatricians. Physicians in Europe and Asia had a higher smoking prevalence than in Oceania. The smoking prevalence among physicians has decreased over time. Male physicians had a higher smoking prevalence. Age did not influence smoking prevalence. Conclusion: Prevalence of smoking among physicians is high, around 21%. Family practitioners and medical students have the highest percentage of smokers. All physicians should benefit from targeted preventive strategies.

## 1. Introduction

Smoking is a major public health problem [1]. According to the International Classification of Diseases (ICD−10), tobacco smoking disorder is considered a mental and behavioral disease [2]. Furthermore, according to the World Health Organization, there are about a billion smokers around the world and tobacco kills more than seven million of them per year [1]. Tobacco control has been present in many countries for several years. In 2003, the WHO adopted the Framework Convention on Tobacco Control. Despite their knowledge of the health risks linked to smoking [3], some physicians smoke too [4,5]. Prevalence of smoking among physicians can be a public health issue both for themselves and for patients because they play a key role in combating tobacco use [6]. Indeed, it has been demonstrated that physicians who smoke are less likely to promote quitting smoking to their patients [7,8]. However, the prevalence of smoking among physicians has not recently been systematically reported in the literature. Moreover, some medical specialties may be particularly at risk of smoking, due to workload [9] or work conditions [10], for example. In addition, a country’s culture or wealth can influence the perception of smoking [11,12]. Lastly, the perception of smoking has, historically, changed considerably [13], from a rewarding to a negative image [14]. Although there is a dense literature on the impact of tobacco smoking on health among general population, we did not find any systematic review and meta-analysis on smoking among physicians.

Therefore, we aimed to conduct a systematic review and meta-analysis on the prevalence of smoking among physicians. Secondary objectives were to report physicians’ smoking prevalence depending on their specialties, to investigate differences between countries, changes over time and putative effects of sociodemographic factors.

## 2. Materials and Methods

### 2.1. Literature Search

We reviewed all studies reporting the smoking prevalence among physicians. Eligible articles had to appear on the PubMed, Cochrane Library, Embase, and ScienceDirect databases with the following keywords: “smoking” and “physician” (or “doctor”) and “prevalence”. The search was conducted up to May 2021 (details for the search strategy used within each database are available in Appendix A. Studies could be cross-sectional studies, cohort studies, or clinical trials. The search was not limited to specific years. We limited our search to English or French articles. To be included, studies needed to describe our primary outcome variable, i.e., the prevalence of smoking among physicians. Two authors (Anaïs Besson and Alice Tarpin) conducted the literature searches, reviewed the abstracts, and, based on the selection criteria, decided the suitability of the articles for inclusion and extracted the data. When necessary, disagreements were solved with a third author (Frédéric Dutheil). We followed the Preferred Reporting Items for Systematic Reviews and Meta-Analyses (PRISMA) guidelines [15].

### 2.2. Data Extraction

The primary outcome analyzed was the smoking prevalence and type of smoking (occasional or regular) among physicians. Secondary outcomes reported medical specialty, continent, study’s period, ex-smoking prevalence, sociodemographic parameters (age, gender, family status, and workplace setting), workload (mean duration week), clinical parameters (body mass index and physical activity behavior), and smoking prevalence among a population control.

### 2.3. Quality of Assessment

We used the Newcastle-Ottawa Scale (NOS) to check the quality of included articles [16]. The maximum score was nine for cohort and ten for cross-sectional studies. Additionally, we also used the Strengthening the Reporting of Observational Studies in Epidemiology (STROBE) for cohort and cross-sectional studies [17], and the Consolidated Standards of Reporting Trials (CONSORT) for randomized studies [18] (Appendix B).

### 2.4. Statistical Considerations

Statistical analysis was conducted using Stata software (v16, StataCorp, College Station, TX, USA). Extracted data were summarized for each study and reported as mean (standard deviation) and count (%) for continuous and categorical variables, respectively. Prevalence of smokers and 95% confidence intervals were estimated using random-effects models assuming between and within study variability (DerSimonian and Laird approach) [19]. Then, we stratified results depending on specialties, continents, and periods of time. Statistical heterogeneity between studies was assessed using forest plots, confidence intervals, and I^2^. The I^2^ statistic is the most common metric to measure heterogeneity and is easily interpretable: heterogeneity is considered low for I^2^ < 25%, modest for 25–50%, and high for >50% [20]. We aimed to conduct a sensitivity analysis by excluding studies not evenly distributed around the base of the metafunnel. We also proposed meta-regressions to investigate putative factors influencing the prevalence of smoking in physicians, such as sociodemographic (age, sex), specialties, continents, and periods of time. Results were expressed as regression coefficients and 95% confidence intervals. Type I-error was fixed at α = 0.05.

## 3. Results

**Figure 1:**
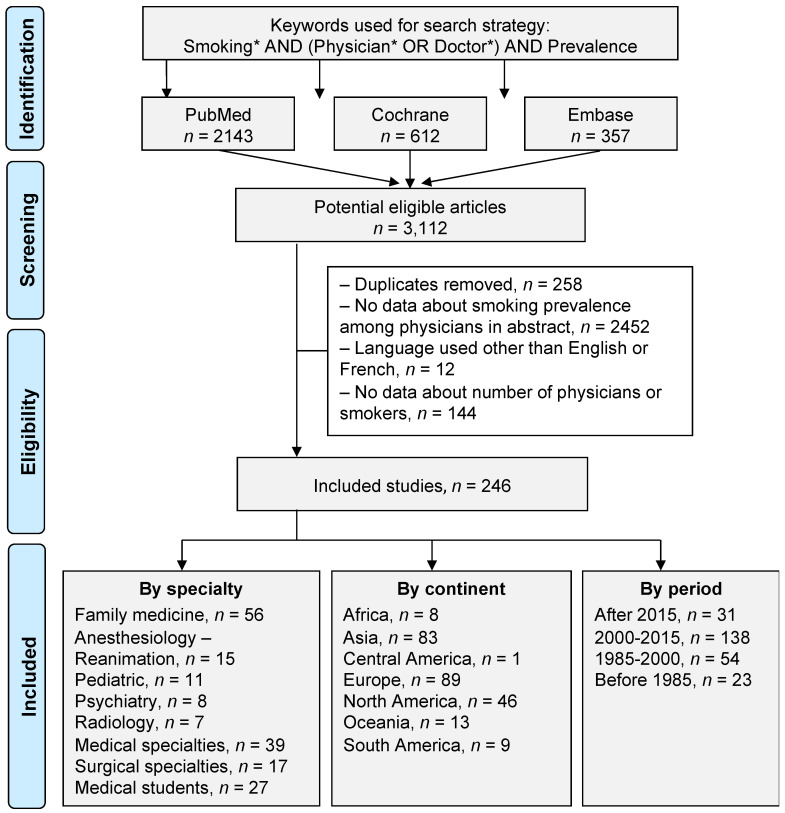
Flow chart. Three databases were asked (PubMed, Cochrane, and Embase). Over 3112 eligible articles, 246 were included. Stratification was performed by specialty, by continent, and by time period. *: details for the search strategy used within each database are available in Appendix A.

An initial search produced 3112 possible articles. Removal of duplicates and use of the selection criteria reduced the number of articles reporting the smoking prevalence among physicians to 246 articles (Figure 1). Main characteristics of the studies are presented in Table 1.

### 3.1. Quality of Articles

**Figure 2:**
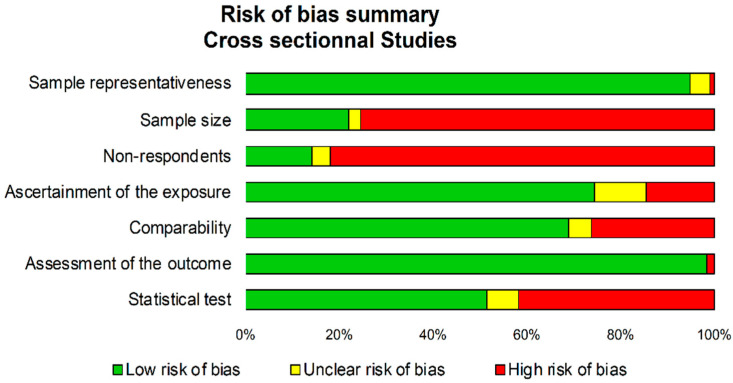
Risk of bias using Newcastle Ottawa Scale composed by seven level of bias assessment.

Using the NOS criteria for cross-sectional studies demonstrated a low risk of bias, except for sample size (not clearly defined in 75% of studies), representativeness (comparability bias in 82% of studies), and statistical tests (not or incompletely described in 49% of studies) (Figure 2). NOS for cross-sectional and cohort assessment are shown in Figure S1 in Supplement. STROBE and CONSORT assessment are shown in Table A1.

### 3.2. Study Designs and Objectives

Most (94%) studies were cross-sectional [7,8,21,22,23,24,25,26,27,28,29,30,31,32,33,34,35,36,37,38,39,40,41,42,43,44,45,46,47,48,49,50,51,52,53,54,55,56,57,58,59,60,61,62,63,64,65,66,67,68,69,70,71,72,73,74,75,76,77,78,79,80,81,82,83,84,85,86,87,88,89,90,91,92,93,94,95,96,97,98,99,100,101,102,103,104,105,106,107,108,109,110,111,112,113,114,115,116,117,118,119,120,121,122,123,124,125,126,127,128,129,130,131,132,133,134,135,136,137,138,139,140,141,142,143,144,145,146,147,148,149,150,151,152,153,154,155,156,157,158,159,160,161,162,163,164,165,166,167,168,169,170,171,172,173,174,175,176,177,178,179,180,181,182,183,184,185,186,187,188,189,190,191,192,193,194,195,196,197,198,199,200,201,202,203,204,205,206,207,208,209,210,211,212,213,214,215,216,217,218,219,220,221,222,223,224,225,226,227,228,229,230,231,232,233,234,235,236,237,238,239,240,241,242,243,244,245,246,247,248,249,250]. However, twelve were cohort [251,252,253,254,255,256,257,258,259,260,261,262] and two were clinical trials [263,264]. Every one of the included 246 studies described smoking prevalence among physicians. The main aim of examining smoking prevalence among physicians was reported in most studies (*n* = 117) [24,26,27,29,32,33,35,36,37,38,39,40,41,44,45,48,49,53,54,55,56,58,59,68,69,72,73,76,78,82,84,85,87,90,91,92,93,94,95,97,98,99,102,103,104,107,108,110,115,119,120,121,126,131,132,133,135,136,137,138,141,142,144,145,146,147,148,149,151,154,156,157,158,160,161,163,164,165,167,169,170,171,172,174,176,178,179,180,182,183,185,187,189,190,191,192,193,194,196,197,199,201,202,203,204,207,211,213,218,224,225,227,231,239,241,242,243]. Fifteen studies also aimed to assess the use of other substances in physicians [29,37,93,94,95,117,118,124,141,161,165,171,193,239,248]. Other outcomes presented were demographic characteristics and health status of physicians in 58 studies [22,23,30,31,42,43,46,57,60,63,64,65,66,74,75,80,88,89,96,100,106,114,116,123,125,128,129,130,134,139,143,153,155,162,176,177,181,184,195,198,208,223,238,239,249,251,252,253,254,255,256,257,258,259,260,261,263,264], the evaluation of smoking cessation counselling among physicians in 50 studies [25,26,28,33,35,40,55,61,62,67,70,79,86,92,97,99,101,104,105,111,136,142,144,145,152,157,168,183,189,190,191,197,201,203,204,205,209,210,212,214,218,219,234,235,236,241,242,244,247,250], the attitude of physicians towards prevention and promotion of a healthy lifestyle in seven studies [122,140,206,208,220,222,228], the knowledge on tobacco effects in 20 studies [24,33,39,44,50,53,61,62,78,91,98,119,154,178,199,204,216,217,240,247], and the examination of the link between smoking habits of physicians and their practice of providing minimal smoking cessation advice in 26 studies [7,8,21,47,58,81,83,109,112,127,150,159,175,186,187,188,208,211,213,224,225,232,237,244,246,262]. Finally, the primary outcome was not clearly defined in 16 studies [34,51,52,71,77,113,166,173,200,215,221,226,229,230,233,245].

### 3.3. Recruitment of Physicians

Physicians were recruited from health centers in 94 studies, either monocentric in 50 studies [21,26,29,30,37,41,42,43,46,49,56,79,80,97,115,120,121,132,133,134,156,162,163,167,170,179,181,182,184,190,193,194,196,202,204,212,217,225,228,229,232,235,244,250,256,257,259,260,263,264] or multicentric in 44 studies [24,39,53,54,72,83,89,90,91,92,98,111,112,117,119,123,126,128,130,131,141,152,168,169,178,197,201,203,205,207,214,216,219,220,224,227,231,236,237,238,242,243,253,261]. They were also recruited from specific lists in 68 studies, either from specific societies in 14 studies [22,40,47,77,78,110,158,180,183,189,221,222,230,240], associations in 23 studies [7,59,67,87,94,95,106,107,108,118,129,135,142,206,209,223,234,239,245,249,251,254,262], medical or specific registers in 22 studies, [23,38,85,88,113,127,139,144,145,154,159,165,173,177,186,192,199,208,210,246,248,258] and lists from ministries of health in 9 studies [33,35,45,99,116,140,146,166,218]. Finally, recruitment procedure was not defined in 84 studies [8,25,27,28,31,32,34,36,44,48,50,51,52,55,57,58,60,61,62,63,64,65,66,68,69,70,71,73,74,75,76,81,82,84,86,93,96,100,101,102,103,104,105,109,114,122,124,125,136,137,138,143,147,148,149,150,151,153,155,157,160,161,164,171,172,174,175,176,185,187,188,191,195,198,200,211,213,215,226,233,241,247,252,255]. Smoking prevalence was also described in non-physicians in 30 studies [24,39,40,52,69,74,79,86,91,99,102,104,105,110,113,125,129,133,135,139,143,151,161,162,176,185,195,197,202,235].

### 3.4. Populations Studied

**Sample size** ranged from 17 [235,237] to 31,208 [64]. In total, 497,081 physicians were included in this meta-analysis.

**Age** of physicians was reported in 89 studies. Overall, the mean age was 41.5 years old (95%CI 38.4 to 44.6), ranging from 20.2 [132] to 59.8 [109] years old (Table 1).

**Gender** was reported in more than half of the studies (*n* = 165) on the total population of physicians, among which 107 studies also reported gender of smoking physicians. The mean number of men was 62% (58 to 65%), ranging from 0 in five studies that included only women, to 100% in thirteen studies that included only men (Table 1).

**Specialty** was reported in 96 studies. Family practitioners were the most represented (56 studies, *n* = 64,187 physicians), followed by medical students (27 studies, *n* = 28,564), medical specialties (39 studies, *n* = 15,538), anesthesiologists (15 studies, *n* = 3329), surgical specialties (17 studies, *n* = 2395), pediatrics (11 studies, *n* = 1847), psychiatrists (8 studies, *n* = 1393), and radiologists (7 studies, *n* = 1193) (Table 1).

**Location** of studies was always reported. Most studies were conducted in Europe (89 studies, *n* = 20,509), followed by America (56 studies, *n* = 126,615), Asia (83 studies, *n* = 104,325), Oceania (13 studies, *n* = 59,609), and Africa (8 studies, *n* = 2023) (Table 1).

**Other variables** were less well described. Family status was reported in 29 studies [22,42,45,74,79,85,89,92,93,94,103,105,112,121,125,128,130,137,143,161,174,179,195,199,229,244,250,257,264], workplace was the focus in 42 studies (most worked in public sectors) [22,28,40,47,58,61,75,79,88,94,96,104,105,125,130,133,134,135,137,148,149,150,155,161,163,172,174,180,185,186,189,199,203,218,219,223,226,229,234,239,240,242], working hours per week was reported in 8 studies (ranging from 37 [25] to 79 [80] hours per week) [22,25,58,75,80,143,195,213], seniority of physician was reported in 14 studies (ranging from 6.5 [205] to 20.8 [28] years ago) [25,28,36,58,78,96,126,130,137,140,150,184,204,205], BMI in 17 studies (ranging from 21 [260] to 27.7 [257] kg/m^2^) [23,30,42,66,80,88,96,130,139,140,141,244,249,255,257,259,260], and physical activity in 24 studies (most physicians were active) [30,42,46,57,75,88,89,96,100,121,122,123,125,129,130,134,139,140,143,195,238,239,255,257]. 

### 3.5. Smoking Assessment

Most studies used a self-administered questionnaire (postal and email) (209 studies) [7,8,21,22,23,24,25,26,27,28,29,30,31,32,33,36,37,38,39,40,41,42,43,44,45,46,47,48,49,50,51,52,53,54,56,57,59,60,61,62,63,64,65,66,67,68,69,70,71,72,75,76,77,78,79,80,81,82,83,84,85,86,87,88,90,91,92,93,94,95,96,98,99,102,105,106,107,108,109,111,112,114,116,117,118,119,120,121,122,123,124,125,126,127,128,129,130,131,132,133,134,135,137,139,140,141,142,143,144,145,146,147,148,149,151,153,154,157,158,159,160,161,162,163,164,165,166,167,168,169,170,171,172,173,174,176,177,178,179,180,181,182,184,185,186,189,190,192,193,194,195,196,197,198,199,201,202,203,205,206,207,209,210,211,212,213,214,215,217,218,219,220,221,222,223,224,225,227,228,229,230,231,232,234,235,236,237,238,239,240,241,242,244,245,246,248,249,250,251,252,253,254,255,256,257,259,260,262,263,264]. Other studies collected data by interview (11 studies) [35,55,100,101,150,155,156,175,191,208,261], interview and postal (7 studies) [58,73,74,115,183,187,188], phone (9 studies) [89,103,104,136,152,204,216,226,233], and phone and postal (1 study) [98]. The data collection method was unclear in nine studies [34,97,110,113,138,200,243,247,258]. The definition of smoking used was not explained in most (96.2%) included articles. In eight studies, smoking was defined by one cigarette per day [24,93,142,156,162,202,203,220]. In five studies, a smoker was defined as a person who had smoked at least 100 cigarettes or an equivalent amount of tobacco in their lifetime [24,138,178,202,261]. Two studies specified whether smokers were cigarette, pipe, or cigar smokers [114,115]. Around half of the studies reported the prevalence of ex-smokers (135 studies, *n* = 47,688) (Table 1). As with smoking, the definition of ex-smoking was not explained in most (95.2%) included articles. In five studies, an ex-smoker was defined as someone who stopped smoking completely for at least 3 [220], 6 [142,261], or 12 months [24,224]. Publication occurred within 2 years of data collection for 31% of studies, within 2 to 5 years for 45%, and more than 5 years for 10%—and was not reported for 14% of studies. Most studies were published between 2000 and 2015 (138 studies, *n* = 232,323), followed by studies published between 1985 and 2000 (54 studies, *n* = 85,402), after 2015 (31 studies, *n* = 94,637), and before 1985 (23 studies, *n* = 84,719). Studies ranged from 1954 [63] to 2021 [243,247] (Table 1).

### 3.6. Meta-Analysis on the Smoking Prevalence among Physicians

**Figure 3:**
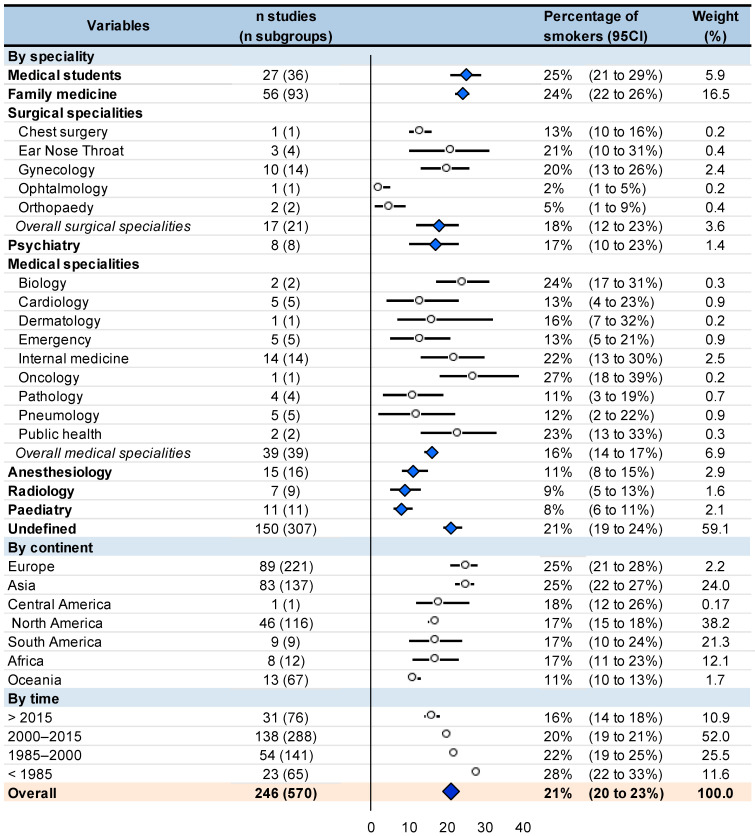
Meta-analysis on prevalence among physicians stratified by specialty, continent, and time. Results are expressed in percentage from 0 to 100. Bold represent a stratification or overall result.

The smoking prevalence among physicians was around 21% (95CI 20 to 23%). Stratified by specialty, prevalence of smoking was 25% (21 to 29%) in medical students, 24% (22 to 26%) in family practitioners, 18% (12 to 23%) in surgical specialties, 17% (10 to 23%) in psychiatrists, 16% (14 to 17%) in medical specialties, 11% (8 to 15%) in anesthesiologists, 9% (5 to 13%) in radiologists, and 8% (6 to 11%) in pediatrics. Stratification by continent showed the prevalence of smoking in physicians ranging from 11% in Oceania to 25% in Europe and Asia. The smoking prevalence among physicians decreased over time: 28% (22 to 33%) before 1985, 22% (19 to 25%) between 1985 and 2000, 20% (19 to 21%) between 2000 and 2015, and 16% (14 to 18%) after 2015. All I^2^ were extremely high within each stratification, i.e., >99%, except two I^2^ that were at 86 and 94% (Figure 3). 

### 3.7. Meta-Regressions

**Figure 4:**
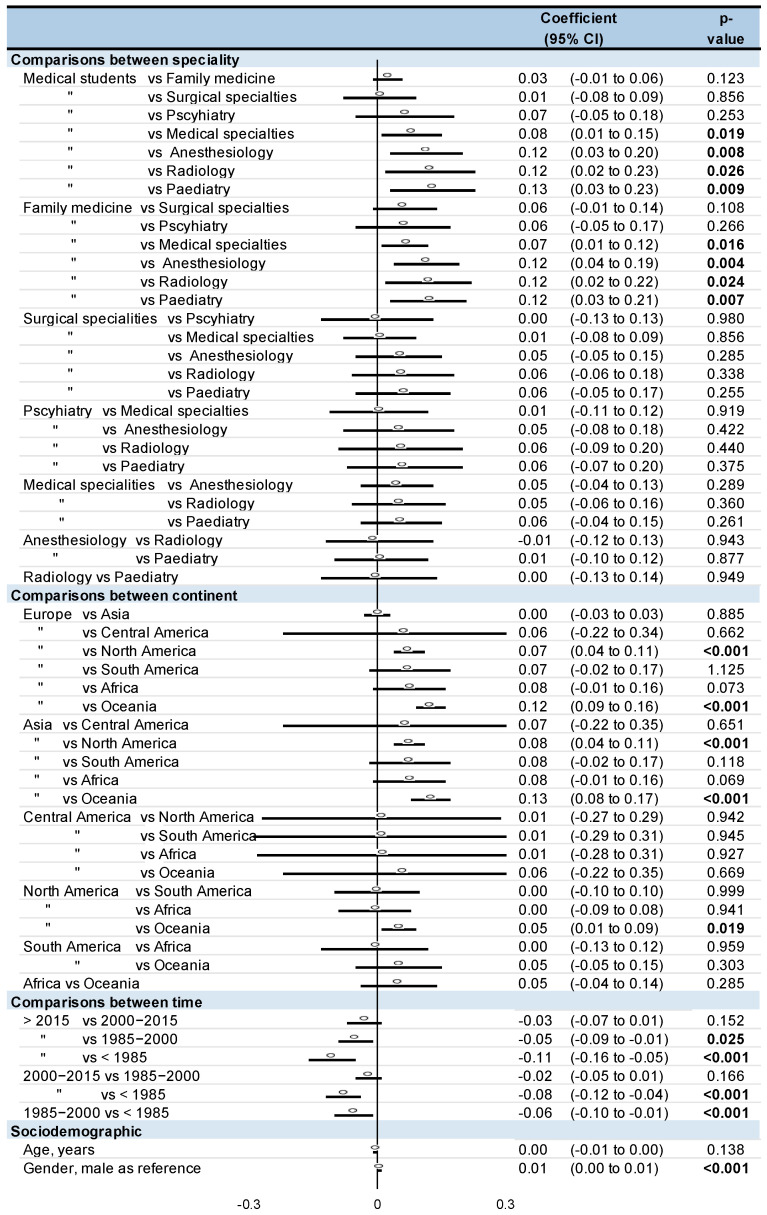
Meta-regressions. “means ‘same as’ the line above. Bold represent a stratification.

Family practitioners and medical students had a higher smoking prevalence than anesthesiologists (Coefficient 0.12, 95CI 0.04 to 0.19, and 0.12, 0.03 to 0.20, respectively), pediatrics (0.12, 0.03 to 0.21 and 0.13, 0.03 to 0.23), radiologists (0.12, 0.02 to 0.22, and 0.12, 0.02 to 0.23), and other medical specialties (0.07, 0.01 to 0.12, and 0.08, 0.01 to 0.15). For comparisons between continents, physicians in Europe and Asia had a higher smoking prevalence than in North America (0.07, 0.04 to 0.11, and 0.08, 0.04 to 0.11, respectively) and Oceania (0.12, 0.09 to 0.16, and 0.13, 0.08 to 0.17). Smoking prevalence in North America was also significantly higher than in Oceania (0.05, 0.01 to 0.09). Lastly, smoking prevalence was the highest before 1985 (0.06, 0.01 to 0.10 vs. between 1985 to 2000; 0.08, 0.04 to 0.12 vs. between 2000 to 2015; 0.11, 0.05 to 0.16 vs. after to 2015). Moreover, the smoking prevalence between 1985 and 2000 was higher than after 2015 (0.05, 0.01 to 0.09). Male physicians had a higher smoking prevalence than women (0.01, 0.00 to 0.01). Age did not influence smoking prevalence (Figure 4). Insufficient data precluded other meta-regressions. 

### 3.8. Sensitivity Analyses

Funnel plots of meta–analyses analyzing for potential publication bias are presented in Figure 2. Due to the huge heterogeneity (most I^2^ being >99%), we did not reperform meta–analyses after the exclusion of studies that were not evenly distributed around the base of the funnel. Lastly, we performed all aforementioned analyses on the prevalence of ex-smokers. The prevalence of ex-smokers among physicians was around 23% (95CI 21 to 25%). Psychiatrists also had a high prevalence of ex-smokers (29%, 19 to 40%), followed by other specialties. Contrary to meta-analyses on current smokers, medical students had a low rate of ex-smokers (11%, 6 to 17%). Interestingly, if prevalence of current smokers was similarly high in Europe and Asia (25%), there was greater prevalence of ex-smokers in Europe (25%, 21 to 29%) than in Asia (17, 14 to 20%) (*p* < 0.001). North America and South America also had a high prevalence of ex-smokers (27%, 20 to 34%; and 26%, 14 to 38%, respectively), whereas Africa had a low prevalence of ex-smokers (8%, 6 to 10%). The prevalence of ex-smokers decreased in similar proportions over time: 31% (26 to 35%) before 1985, 24% (18 to 30%) between 1985 and 2000, 22% (19 to 24%) between 2000 and 2015, and 21% (14 to 28%) after 2015 (Figure 3 and Figure 4).

## 4. Discussion

The main findings were that the prevalence of smoking among physicians is high, around 21%. Family practitioners and medical students have the highest percentage of smokers and should benefit from targeted preventive strategies. Smoking in physicians is a public health issue that is common, both in developed and developing countries, even if quitting smoking is higher in developed countries. Positively, the prevalence of smoking decreased over time.

### 4.1. Smoking among Physicians: A Public Health Issue

Surprisingly, prevalence of smoking among physicians is high, which may seem unlikely because they should be an example for their patients and should know the health risks linked to tobacco [21]. This said, even if there is no study determining whether being a physician is a risk factor for smoking compared to the general population, they seem to follow similar trends and are highly concerning [265]. Literature shows that disadvantaged populations smoke more than others [266]. In some way, physicians can also be considered as disadvantaged due to their cumulative risk factors for smoking. They face a huge workload, working over 55 h a week [22]. Stress at work could play a major role in their smoking habits [22]. Overload of stress can even contribute to depressive disorders and high risk of suicides, that are also risk factors of smoking [267,268]. They also work nightshifts [43], disrupting the circadian rhythm that can heighten smoking behavior [269]. Moreover, despite the consequences for themselves, physicians who smoke are less likely to promote quitting smoking for their patients [7,21]. Therefore, there is a need to tackle physicians smoking behavior both for themselves and their patients. Smoking in physicians must be considered as a major public health problem. Alarmingly, even our massive search did not find governmental actions for quitting smoking in physicians. In the research, we found several randomized controlled trials on strategies for smoking cessation in homeless people [270,271], but none in physicians. 

### 4.2. Depending on Specialties

The smoking prevalence was higher among medical students and family practitioners. For medical students, the high prevalence of smoking may be explained due to the stress of hard academic studies [272]. Moreover, medical students can have high risk-taking behaviors such as partying and tobacco consumption [273,274]. The high prevalence among family practitioners might be explained by several putative factors such as workload [22], stress [22], and lack of cohesive teamwork [275]. Workload and stress have been shown in the literature as an important risk factor of smoking [22]. The work environment, such as the lack of cohesive teamwork, is a risk factor of depression and drug use [275], with depression and drug use being linked [60]. Similarly, workload [22] and work stress [22] can also contribute to the high prevalence of smoking in surgical practitioners, who can face legal issues as part of their work [276]. Experiencing judgement in court and repeated trials could promote depression and, in turn, smoking [276]. For psychiatrists, contributing factors of smoking could be the fact that they are routinely faced with traumatic experiences [277], incurable diseases [278], and breaking bad news to patients [278]. Conversely, pediatrics smoked the least, probably because they most often deal with common and curable diseases [279]. Moreover, pediatricians are predominantly women [280]. Although we found a higher prevalence of smoking among women, the literature described a lower rate of smoking among women compare to men in the general population [45,243]. A common characteristic of all specialties is that smoking cessation training during medical studies was poor or not important enough [62,70]. This may contribute to the high prevalence of smoking among physicians. Considering that nearly all physicians will encounter smoking patients, improving smoking cessation training during their studies could help both their patients to quit smoking, as well as the physicians themselves.

### 4.3. Depending on Continents

Smoking prevalence was not homogeneous between continents. Europe and Asia were continents where the smoking prevalence among physicians was the highest. Conversely, Oceania was the continent where the smoking prevalence among physicians was the lowest. This heterogeneous prevalence was probably in line with tobacco culture [281] and tobacco marketing [281] in many countries. Tobacco culture in Europe was brought by Christopher Colombus in the 16th century [282], firstly as a luxury product [13]. But during the 20th century, tobacco became accessible for all and became a trendy product [13]. Then, in developed countries, tobacco became undesirable [283]. Recent literature shows that tobacco marketing targeted more poor countries [284]. India is the country on the Asian continent with the poorest population, and represents about 17% of the global population [285,286,287]. Moreover, tobacco control is less important in Asia [288]. Studies conducted in Oceania were conducted in rich countries, i.e., Australia and New Zealand, that may be the two countries with the strongest anti-smoking policy [289]. In those two countries, the price of cigarettes is among the most expensive. The increasing taxes aided the decrease in prevalence of smoking, which can be an easy reproducible preventive strategy in other countries [290]. In 2012, Australia was the first country to use plain cigarette packaging [291,292]. A national tobacco campaign in Australia showed the benefits of stopping smoking rather than the negative effects of tobacco [293]. In New Zealand, smoking is prohibited in motor vehicles carrying children under the age of 18 [294]. Finally, except in those two countries that manage smoking, smoking is still a major public health issue worldwide, both in developed and developing countries.

### 4.4. With a Time Effect

The smoking prevalence among physicians decreased overtime. We showed that physicians’ smoking prevalence has decreased since 1985. The knowledge of the health risks of tobacco during the 1970s changed tobacco from a positive to a negative image [13]. The most recent studies (after 2015) showed that the prevalence of tobacco in physicians continued to decrease. Universally, this decrease was probably related to the tobacco control implemented by the WHO [295], such as a tobacco free-day since 1987 [296]. The WHO Framework Convention on demand and supply reduction [295] probably played a major role in the tobacco consumption decline. Since 2003, European directives limit physicians’ work to 48 h per week [297,298], which may have lessened the stress of physicians. The development of new technologies has encouraged public health advocates to adapt to target a younger cohort, such as the creation of a mobile app for assisting smokers [299], sending emails [300], or sending mobile text messaging [300]. Even if the number of studies on the toxicity of electronic cigarettes remains low, it seems interesting to help with smoking cessation [301]. In Canada, mailed distribution of free nicotine patches seems beneficial, particularly among the financially disadvantaged [302]. In France, nicotine substitutes are reimbursed at 65% by the National Health system as of January 2019 [303]. Our meta-analysis showed that many studies were carried out between 2000 and 2015, probably to assess the effectiveness of tobacco control [295]. Interestingly, preventive strategies sometimes took advantage of context. With the COVID pandemic, Santé Publique France led a digital campaign and special operation to promote the tobacco control [304], based on the fact that tobacco aggravates COVID’s symptoms [305]. That said, the decrease in smoking prevalence could continue in the coming years.

### 4.5. Other Influencing Variables

Male physicians always smoked more than women, probably because of social habit [306]. There was no significant effect of age on the smoking prevalence of physicians, however, smoking prevalence among the general population decreases with age [307,308]. Insufficient data precluded further analyses on putative influencing factors such as physical activity, BMI, number of hours worked per week, workplace setting, or family status. For example, lower physical activity and higher waist circumference were associated with tobacco consumption [309]. Leisure physical activity of physicians is low [310], which can be limited by their workload [310]. Low levels of physical activity also contribute to burnout [311], that, in turn, increases smoking [22]. No study compared smoking prevalence based on the type of practice (public or private practice). Even if being divorced or separated is a risk factor for smoking in the general population [312,313], the influence of family status in physicians has not been reported. To our knowledge, smoking prevalence of physicians was never compared with smoking prevalence of the general population. Physicians also have protective factors of smoking. For example, their level of study is above the baccalaureate [307], their income is higher than the average population [307], and they are most likely to know tobacco risks [21]. Considering that physicians combine risk and protective factors of smoking, comparisons with the general population may be of particular interest to target appropriate preventive strategies.

### 4.6. Limitations

Our study has some limitations. We conducted our meta-analyses on only published articles, so our results were, theoretically, exposed to a publication bias. We included only studies reporting physicians’ smoking prevalence and only studies written in English or French, so our results were, theoretically, exposed to a selection bias. Most cross-sectional studies included in our meta-analysis described a bias of self-report. Data were collected by self-administered questionnaire, not always anonymously. Thus, the reporting of smoking might have been underestimated by physicians. Another limitation could be the number of different studies included and the number of physicians included. Although we did not find any double inclusion, it could be possible that some physicians were included twice, creating an overlap that might introduce some bias. Our meta-analysis also had limitations on the definition of smoking. In fact, the definition used to define regular smokers, occasional smokers, or former smokers was different between studies and was rarely detailed. Therefore, the meta-analysis inherited the limitations of the individual studies of which they were comprised: varying quality of studies, multiple variations in study protocols, and evaluation. Comparisons between specialties might suffer from a bias, such as a different number of physicians within each specialty. Moreover, our meta-analysis had a lot of studies with undefined specialties. Similarly, some authors suggested that the medical field was mainly dominated by the male gender and reported a poor status integration of women physicians within the profession [314]. Comparisons between continents or time period might also suffer from a different number of studies within each continent or each period; however, our review provided a massive sample of nearly half a million physicians promoting generalizability of our results.

## 5. Conclusions

We found that the prevalence of smoking among physicians is high, around 21%. There is an important heterogenicity between specialties, continents, and periods of time. Despite family practitioners and medical students being the heaviest smokers, all physicians should benefit from targeted preventive strategies. Smoking in physicians is a public health issue that is common, both in developed and developing countries, even if quitting smoking is higher in developed countries. Positively, the prevalence of smoking decreased over time, but pursing tobacco control is necessary.

## Figures and Tables

**Table 1:** Characteristics of included studies.

Study	Country	GDP per Capital	Period of Data Collection	Physicians				Prevalence	(%) of	Smokers		% Men among Smokers
				*n*	% Men	Age	Speciality	Regular Smokers	Occasional Smokers	Former Smokers	Total (Regular & Occasional)	
Aaro 1977	Norway	6812	1952–1974	3544			Not defined				58.3	92.5
Abdullah 2006	China	1149	2002	757	78.2		Not defined			2.1	4.3	
Aboyans 2009	France	41,508	2007	371		49.8	Cardiology			32.4	8.1	
Akvardar 2004	Turkey	6041		319			Study, Others				37.6	
Al Alwan 2013	Saudi Arabia	16,094	2009	100			Not defined			7	12	
Alarjan 2015	Jordan	4096		162			Not defined				34.0	
Al-Khateeb 1990	Bahrain	7959	1988	301			Not defined			8.6	60.1	
Al-Lawati 2009	Oman	16,784		1079			Family medicine, Biology, Others				22.2	97.5
Allan 1976	Australia	7475		1153			Family medicine, Radiology, Pathology, Anesthesiology-Reanimation, Gynecology, Others				14	
Al Shahrani 2021	Saudi Arabia	23,338	2018	290	59.7		Not defined				34.8	70.3
Amara 2008	Morocco	2885	2007	75		44.4	Pneumology	37.5	62.5	12	10,7	
Amte 2015	India	1606		242	84.7	37.9	Not defined	36.4	63.6		13.6	
An 2004	USA	36,334	2000	750	68.9		Not defined			16.9	1.5	
Arnetz 1988	Sweden	24,189		66	100	43.9–46.8	Family medicine, Others			23.3–33.3	22.7	100
Aryayev 2014	Ukraine	3105		150	31.3		Family medicine, Paediatry, Study				42	61.9
Baltaci 2014	Turkey	10,672	2010–2011	1233	57.1	38.94	Not defined			14.8	34.1	
Baptista 1993	Venezuela	2368	1990	191	49.7	31	Not defined				20.9	
Barengo 2004	Finland		1990–2001	4546	51.4		Not defined	38.1	61.9		16	
Barengo 2005	Finland	24,913	2001	707			Family medicine				12.7	
Barnoya 2002	Guatemela	1702	2002	174	56.9		Not defined			35	17.8	56.9
Basnyat 2000	UK	28,015	1998	314	97.8	51	Not defined			37	10.2	97.8
Basu 2011	India	1346	2010	182			Study	85.5	14.5		30.2	
Behbehani 2004	Bahrain, Kuwait		2000–2001	1440	66.5	44.6–44.7	Not defined	62.1	37.9	14.3–15.9	17.2	92.7
Belkić 2007	Serbie	2150	2002–2004	112	0	48.9	Not defined			12.5	31.3	0
Belkić 2012	Serbie	2150	2002–2004	191	42.9		Not defined				30.4	39.7
Bener 1993	Kuwait, United Arab Emirates		1990–1992	527			Not defined			12.7–13.5	37	
Borgan 2014	Bahrain	24,737	2013	148	33.8	45	Not defined			3.9	10.1	80
Bortz 1992	USA	25,493		126	77.8		Not defined			22.2	1.6	100
Bostan 2015	Turkey	10,672	2010–2011	699		38.7	Pneumology			19.5	9.9	
Bourke 1972	Ireland	1152	1967–1969	1580	86		Not defined			23.6–42.5	45.4	91.8
Braun 2004	USA	38,166	2002	1067	68	46	Not defined			18.6	1.3	
Brenner 1996	Germany	26,334	1992–1993	696	52.6		Study			11.9	23.7	64.8
Brink 1994	USA	23,954	1990	132			Not defined				1.6	
Brotons 2005	Croatia, Estonia, Georgia, Greece, Ireland, Malta, Poland, Slovakia, Slovenia, Spain, Sweden		2000	2082	40	35.6–51.4	Family medicine				16.4	
Burgess 1970	USA		1963–1968	1863			Not defined				27.3	
Burgess 1978	USA	6741	1973	1234	95.1		Family medicine, Internal medicine, Paediatry, Psychiatry, Radiology, Pathology, Gynecology, Orthopaedy, Anesthesiology-Reanimation, Ear Nose Throat, Others			35.4	19	95.7
Cao 2011	USA			19,705	100	58.3	Not defined			41.7	6.7	100
Carlos 2020	Spain	30,389	2018	890	52	51.7	Not defined			24	16.5	
Ceraso 2009	China	2099	2006	103	100		Not defined				49.5	100
Chaudhry 2009	Pakistan	874	2006	120	75		Not defined				23.8	100
Cheng 1990	Hong Kong	9071	1987	133	81.2	25	Not defined	33.3	66.7		15.8	90.5
Coe 1971	USA	862	1967	1572			Family medicine, Internal medicine			31.3–34.9	29.6	
Cofta 2008	Poland	14,001		117			Not defined	81.8	18.2	10.3	9.4	
Das 2013	India	1462	2011	600	67		Study				14.5	
Davies 1989	UK	13,119	1987	94	83		Not defined			26	3	
De Col 2010	France	45,334	2008–2009	332	67.8	50.7	Family medicine	57.4	42.6	34	18.4	
Dekker 1993	Netherlands	17,176	1989	619	70.6		Family medicine, Study, Others			29.6	83.6	
De Oliveira 2013	USA	52,782		1480			Study				6.9	
Desalu 2009	Nigeria	1384	2008	436	75.7	30.6	Not defined				17.7	
Djalalinia 2011	Iran	1892	2002–2003	5140	74	35	Family medicine				11.8	100
Dodds 1979	Australia	7763	1977	275	84.7		Not defined			30	21	87.9
Doll 1954	UK		1951	24,389	100		Not defined				87.3	100
Doll 1964	UK		1951–1958	31,208	100		Not defined			15.3–26.6	61.8	100
Doll 1994	UK		1951–1990	10,812	100		Not defined			13–60	39.9	100
Doll 2004	UK	19,709	1978	12,669	100		Not defined			46.3	30.5	100
Easton 2001	USA	26,464	1993–1994	1590	0	41	Not defined			19.9	3.5	0
Easton 2001	USA	26,464	1994	1397	0	42	Not defined			21.5	4.7	0
Edwards 2008	New Zealand	26,671	2006	10,200	60.2		Family medicine, Study, Radiology, Gynecology, Anesthesiology-Reanimation, Others				3.5	67.6
Edwards 2018	New Zealand	42,949	2013	12,684	55.7		Family medicine, Study, Radiology, Gynecology, Anesthesiology-Reanimation, Others			12.1	2.1	61.6
Fadhil 2007	Bahrain	17,959	2005	120	35.8	36.5	Family medicine			10	24.2	
Fanello 1990	France		1973–1987	2718			Family medicine				44.6	
Fathi 2016	Iran	7833	2012–2013	225			Not defined				21.3	
Fowler 1989	UK	15,987	1988	3240	77		Family medicine			33	13.5	
Franceschi 1986	Italy	7964	1985	709			Family medicine, Internal medicine, Public health, Others			14–26	31.3	
Frank 1998	USA	27,777	1994	4501	0	42.2	Not defined			18.6	3.7	0
Frank 2009	Canada	44,545	2007–2008	3213	66		Not defined	57.1	42.9		14	
Freour 2011	France	41,575	2009	337			Not defined			30.6	12.5	
Garfinkel 1976	USA		1959–1972	8503			Not defined				32.7	
Glavas 2003	Greece	18,478		119	76.5		Not defined				37	
Grossman 1999	Costa Rica	2828	1993–1994	216	70.8	41	Not defined			40	19.4	61.9
Gunes 2005	Turkey	3660	2002	257	77.8	31.3	Not defined	81.3	18.8		37.4	
Gupta 2013	USA	47,100	2009	177	41.2		Internal medicine, Paediatry, Others, Emergency	99.6	0.4		0.6	
Hallett 1983	UK		1980	385			Family medicine			44.4	27.8	
Hamadeh 1999	Bahrain	10,131	1994	122	52.5		Family medicine	66.7	33.3	14.8	17.2	81
Han Zao Li 2008	China	1753	2005	326	59.2		Not defined				42	81.8
Hay 1976	New Zealand		1963–1972	763	0	45	Family medicine, Psychiatry, Anesthesiology-Reanimation, Others	93.6	6.3	22.3–26.9	20.4	
Hay 1998	New Zealand		1976–1996	7335	68.5		Family medicine, Radiology, Gynecology, Anesthesiology-Reanimation, Others				5	71.5
Heloma 1998	Finland	26,009		332	84.3	42.6	Not defined	40	60	20.2	24.1	
Hensrud 1993	USA	26,387		389			Not defined			37.3	9	97.1
Hepburn 2000	USA	31,573	1997	150			Family medicine				11	
Heponiemi 2008	Finland	41,188	2006	2652	40.8	53	Not defined				12.4	
Hidalgo 2016	Brazil	13,246	2011	182	56.6		Not defined			12.3	5.5	
Hill 1997	USA	27,777	1994	121	53.7		Not defined				4.1	
Hodgetts 2004	Bosnia Herzegovina	1769	2002	112			Not defined			13.6	39.3	
Hoseainrezae 2013	Iran	6111		252			Not defined			7.1	9.5	
Huang 2013	China	5618	2011	720	100		Not defined	60	40		25.7	100
Hughes 1991	USA	20,039	1987	1733	69.4	30	Not defined				5.3	
Hughes 1992	USA	22,857	1989–1990	5426	82.4		Not defined				3.9	
Hughes 1999	USA	22,857	1989–1990	5418			Family medicine, Internal medicine, Emergency, Pathology, Paediatry, Psychiatry, Anesthesiology-Reanimation, Gynecology, Others				14.3	
Hung 2013	USA	48,467	2010	1000	68.5	45.3	Family medicine				4	
Hussain 1993	UK	19,901	1991	1069			Not defined				5	
Içli 1992	Turkey	2736	1991	200			Study				34	
Innos 2002	Estonia		1982	3673	23.7		Not defined			13.1	21.3	45.6
Jacot Sadowski 2009	Swiss	41,376	2002	1856	78.8		Not defined	40.8	59.2		17.6	
Jiang 2007	China	1509	2004	3552	55.1		Not defined			2.7	22.9	98
Jiménez-Ruiz 2015	Spain	29,462	2014	416	59.4		Not defined	80.4	19.6	38	11.1	
Jingi 2015	Cameroon	1381	2012	65	69.2	39.1	Family medicine				12.3	100
John 2003	Germany		1989–1999	2509			Not defined			18.9–22.5	20.4	
Joossens 1987	Belgium	8846	1983	2157			Not defined			33	32	
Josseran 2000	France, Netherlands, Spain, UK, Greece, Brazil		1992–1997	16,788			Family medicine, Study, Others				9.5	
Josseran 2005	France	24,974	1998	2073	79.3	45.1	Family medicine			45.5	32.1	83.6
Julião 2013	Brazil	8598	2009	515	66.8	45.3	Not defined			23.3	5.8	
Kaetsu 2002	Japan		1983–1990	5312	95.7		Not defined				36.6	99.3
Kaetsu 2002	Japan	10,425	1983	4190	95.6		Not defined				41.9	99
Kai 2008	Japan	37,217	2005	1063			Anesthesiology-Reanimation, Chest surgery			30	12	
Kaneita 2010	Japan		2000–2008	10,890	66.4		Not defined				16.1	88.2
Kawahara 2000	Japan	38,437	1996–1997	709	91.8	54.7	Not defined			46.3	26	98.4
Kawakami 1997	Japan	39,269	1994	323	84.8	59.8	Not defined			46.1	21.1	95.6
Kawane 1993	Japan	24,813	1989	6224			Pneumology			39.4	24.8	
Kono 1985	Japan	920	1965	5446	100		Not defined				67.8	
Kotz 2007	Netherlands	29,204	2002–2003	1180		45.9–48.3	Family medicine, Cardiology, Pneumology			24.8–29.7	6.6	
Lam 2011	China	2099	2006	504	100		Not defined				46.2	100
La Vecchia 2000	Italy	21,998	1999	501	76.6	45	Not defined	87.7	12.3	26.5	27.5	
Lefcoe 1970	UK	2348		310	100	45.7	Not defined			19.7	51.9	100
Legnini 1987	USA	18,237	1985	266			Public health, Internal medicine, Psychiatry, Others			17.1–37	21.1	
Lindfors 2009	Finland	37,703	2004	328	53.4	47	Anesthesiology-Reanimation				16.5	
Linn 1986	USA	17,134	1984	211	91		Not defined				4	
Lipp 1972	USA	5234	1970	1061			Study				17	
Lipp 1972	USA	5609	1971	1314			Not defined			40	21	
Magee 2017	Georgia	4739	2014	86			Not defined			14	18.6	
Malik 2010	Pakistan	1007	2009	234	69.7		Not defined				37.2	94.3
Manson 2000	USA	14,434	1982	21,068	100		Not defined			39.2	11	100
Mappin-Kasirer 2020	UK		1951–2016	29,737	100		Not defined			14.9–68.4	8.4–67.2	100
Marakoğlu 2006	Turkey	8035		363	69.1	34.2	Not defined			9.9	28.7	85.6
Márk 1998	Hungary	4495	1995	170	62.9		Not defined				25.9	
Mathavan 2009	India	1102		1433	65.7		Not defined	66.5	33.5		11.9	100
McAuliffe 1984	USA	14,439	1982	134			Not defined				6	
McEwen 2001	UK	28,383	1999	303	68		Family medicine				4	
McGrady 2007	Ireland	47,631	2004	650		46.1	Family medicine			15.2	4.2	
Mejia 2011	Argentina	5110	2005	235	54.5	45	Gynecology			26.3	35.3	
Merrill 2006	Jordan	2548	2006	251	69.3	45.3	Not defined	63	37	17.5	18.3	84.8
Meshefedjian 2010	Canada		2000–2004	610	55.1		Family medicine			32	7.4	51.1
Mikalauskas 2012	Lithuania	11,837	2009	59		43.8–44.4	Anesthesiology-Reanimation, Others				13.6	
Misra 2004	USA	32,854	1998–2000	254		50.88	Not defined				3.5	
Miwa 1995	Japan	31,465	1992	17	94.1		Cardiology				41.2	
Mohan 2006	India	547	2003	229	66.4	42.7	Not defined			14.4	8.7	100
Mohseni-Bandpei 2011	Iran	5630	2008	223	48.4	42.7	Not defined				13.5	
Moreno 2006	Spain	21,463	2003	147		21.5–39.1	Study, Others			7.3–26.7	47.6	
Mostafa 2017	Egypt	3525	2016	521	64.3		Internal medicine, Dermatology, Paediatry, Gynecology, Others			8.3	21.5	89.3
Movsisyan 2019	Armenia	3607	2015	36	25		Study			0	16.7	100
Mubeen 2008	Pakistan	683	2005	165	43.6	20.16–22.89	Study	37.5	62.5	2.4–3.8	14.5	95.8
Naji 2006	Ireland	32,541	2002	106	61.3		Not defined			22.6	21.7	60.9
Nakládalová 2005	Czech Republic	8033	2002–2004	370	54.9		Not defined				16.8	62.9
Nardini 1998	Italy	20,665	1995	605	81.3	44	Not defined			34.4	25	
Nawaz 2007	Pakistan	625	2004–2005	1029	44.6	21	Study	63.5	36.5	22.4	11.2	89.6
Nawaz 2008	Pakistan	837	2006–2007	227			Family medicine				36.1	
Ndiaye 2001	Senegal	681	1999	163	78.5	41	Not defined			6.8	27.6	93.3
Nelson 1994	USA		1974–1991	379			Not defined			27.8–32	9	
Ng 2007	Indonesia	1065	2003	447			Not defined				11.6	96.2
Nollen 2004	Nigeria	742	2002	373	83.9	33	Not defined				2.9	
Nutbeam 1990	UK	19,096		304	82.6		Family medicine			32	13.8	100
Obeidat 2017	Jordan	4073	2014	104		42.7	Not defined				44.2	
O’Cathail 2013	Ireland	52,105	2009–2010	248			Not defined			20.8–22	8.1	50
Ohida 2001	Japan	38,532	2000	3771	66.3		Not defined				20.3	88.7
O’Keeffe 2019	Ireland	55,413	2014	1746	50.5		Paediatry, Pathology, Psychiatry, Emergency, Anesthesiology-Reanimation, Gynecology, Ophtalmology, Others	27.2	72.8		9.3	61.1
Öztürk 2012	Turkey	11,707		80	88.8		Surgery, Study			10	17.5	100
Pärna 2005	Estonia, Finland		2001–2002	4549	29.9		Not defined	49.1	50.2	19.6–36.9	14.2	48.5
Pärna 2005	Estonia	5345	2002	2668	17.4	47.6	Not defined			19.6	13.3	32.8
Pärna 2017	Estonia		1982–2014	9423	20.2		Not defined	82.5	17.5	13–19.1	14.7	41
Perrin 2006	Armenia	1192	2004	236	43.6	43.5	Not defined	83.8	16.2	10.59	33.9	71.3
Peykari 2010	Iran	6603		5140	74	35	Family medicine	47.8	52.2	6.5	15.9	
Phillips 1968	Canada	3463		1743			Not defined	77.2	22.8	27.2	45.8	
Pillay 2020	India	2005	2018	692	70.4	39	Not defined			8.5	7.7	
Pipe 2009	France, Germany, Greece, Italy, Netherlands, Poland, Spain, Sweden, Swiss, Turkey, UK, USA, Canada, Mexico, Japan, Korea		2006	2836	76	48	Family medicine, Internal medicine				42.3	78.1
Piryani 2004	Pakistan	461	1998	200	71		Not defined				32	93.8
Pizzo 2003	Italy	20,088	2000	526			Family medicine				28.3	
Poanta 2006	Romania	5829		112	35.7	39.5	Not defined				42	46.8
Põld 2017	Estonia		2002–2014	4877	16.9		Not defined	67.5	32.5		10.4	34.3
Põld 2020	Estonia	20,367	2014	2903		54.5	Not defined				5.9	63.7
Polyzos 1995	Greece	11,176	1992	148			Not defined				49.3	
Power 1999	Ireland	26,284	1999	171			Family medicine				16.1	
Ramachandran 2008	India	628	2004–2006	2499	75.2	39	Not defined				8	100
Ranchal 2018	Spain		1986–2016	938			Not defined			3.6–20.1	23.2	
Rankin 1975	Australia	6993		1276	87.6		Not defined			38	14.2	
Ravara 2014	Portugal	23,030	2009	608	37.3	39.1	Family medicine, Study, Others			17.3	20.9	52
Reile 2018	Estonia	20,367	2014	1759	82.4		Not defined				7.9	86.3
Roche 1995	Australia	20,320	1995	1365	53.1	29.8	Not defined				6	53.7
Roche 1996	Australia	21,861	1996	908	46.9	28.7	Family medicine			8.3	4	
Rurik 2008	Hungary	10,286	2004	156	42.9		Family medicine, Others				8.3	23.1
Rurik 2014	Hungary	13,046	2009	208	39.9	55.2	Not defined				5.8	33.3
Saadat 2012	Scandinavia			58	53.4		Anesthesiology-Reanimation				12.1	
Sachs 1983	USA	15,561	1983	567			Pneumology				12	
Saeed 1991	Saudi Arabia	7839		698			not defined				34	
Saeys 2014	Belgium	47,349	2011	626	57	45	Family medicine	50	50	14	8	72
Salgado 2014	Argentina	12,849	2011	1659	26.9		Study	68.7	31.3	51.72	27.3	28.7
Samuels 1997	Israel	19,653		260	74.2	41	Study, Paediatry, Radiology, Others			20	15.8	
Schnoll 2006	Russia	6920		63	82.5	41.3	Oncology			50.8	27	
Scott 1992	USA		1963–1988	8589			Not defined				13.4	
Sebo 2007	Swiss, Finland, Bosnia and Herzegovina, USA		1989–2004	1784	83.6	51	Family medicine, Internal medicine, Paediatry, Cardiology, Others			22.4	12.3	85.5
Seiler 1983	UK	8692	1983	607			Family medicine				19	
Senior 1982	Canada	12,440	1982	88			Not defined				19.3	
Sharma 1988	India		1982–1987	127			Not defined				34.6	
Shi 2010	China	3832	2009	467	54.8		Anesthesiolopgy-Reanimation			10.9	10.1	
Shin 2012	China	4550	2010	17	47.1	39.4	Not defined				29.4	
Shishani 2008	Jordan	3386		87			Not defined				43.7	
Shishani 2011	Jordan	2774	2007	242	86		Not defined			12.4	46.7	
Shkedy 2013	Isreal	36,310		140			Internal medicine, Paediatry, Anethesiology -Reanimation, Ear Nose Throat, Gynecology			10–27.3	15.7	45.5
Siddiqui 2001	Saudi Arabia	8685		20			Not defined			10	20	
Singh 1981	India	186	1977–1978	861			Study, Others				27.5	
Smith 2006	China	1509	2004	286			Not defined			1	15.7	
Smith 2007	New Zealand		1963–1996	22,097			Not defined			19–37	17.9	
Sotiropoulos 2007	Greece	18,478	2003–2005	1284	55.9	38.4	Family medicine, Internal medicine, Biology, Others			13.8	38.6	58.3
Squier 2006	Ukraine	1048	2003	799	35.9	45	Family medicine			21.6	13.9	
Steinberg 2007	USA	38,166	2002	334	70.7	49	Not defined			23	3.3	
Stuyt 2009	USA	47,976	2007	1319	68.8	44.3	Family medicine, Internal medicine, Paediatry, Psychiatry, Emergency, Anesthesiology-Reanimation, Gynecology, Others			1	38.9	75.4
Sundquist 1999	Sweden	32774	1996	1004	46.2		Family medicine				8.4	50
Svärdsudd 2002	Sweden	24,225	1993–1999	974			Family medicine, Paediatry, Internal medicine, Psychiatry, Radiology, Orthopaedy, Ear Nose Throat, Gynecology, Others			3–43	7.5	
Tapia-Conyer 1997	Mexico	5650	1993	3488	66.2	37	Not defined			20.6	26.9	
Tee 2007	Malaysia	5594	2005	481	39.1		Study			2.7	1.7	75
Tessier 1996	France	22,380	1993	730	90	47	Cardiology	51.8	48.2	47	27	
Thankappan 2008	India	541	2003	333	77.5	42.2	Not defined			26.1	10.8	100
Thomas 1986	USA	19,071		106	100	30.2–30.9	Not defined				13.2	100
Thomas 1997	USA		1957–1965	1015		24–27	Study				55.4	
Tomson 2003	Laos	363		151	49.7		Not defined	46.2	53.8		17.2	100
Tong 2010	USA	39,497	2003–2004	1245	69.7		Emergency, Psychiatry, Others			18.4–28.8	3.5	
Torre 2005	USA		1948–1964	1158	91.9		Not defined				51.1	91.7
Tosun 2016	Turkey	11,336	2011–2012	224	65.2	31.71	Not defined				28.1	
Trédaniel 1993	France	16,302	1987	1012	87.5		Family medicine			29.1	36.9	89.3
Ulbricht 2009	Germany			37	51.4	47.5	Family medicine				24.3	
Unal 2017	Turkey		1975–2004	7228	66	43.6	Not defined			22.5	23.9	75.3
Underner 2004	France	24,177	2002	257			Family medicine	60.6	39.4	30.7	25.7	
Underner 2006	France	24,177	2002	257		48	Family medicine	61.2	38.8	31	26.1	
Uysal 2007	Turkey	6041	2004	374	66.8	46	Not defined			29	16	70
Vanderhoek 2013	Canada	52,542	2012	301	48.5	24.4	Study				15.9	
Vanphanom 2011	Laos	710	2007	855	52.9		Not defined	54.4	45.6	18.4	9.2	97.5
Varona 2005	Cuba	2308	1997	121	33.1		Family medicine				18.2	40.9
Viegas 2007	Brazil	4770	2005	830			Not defined	81.7	18.3	22.7	7.2	
Voigt 2009	Germany	34,044	2004–2006	912			Not defined				13.7	
Waalkens 1992	Netherlands	17,398	1989	1085	58.3		Study, Others			14.5–34	28.7	68.8
Wada 2007	Japan	37,218	2005	196	76		Not defined				19.4	
Wada 2011	Japan	40,855	2009	3864	78.3		Not defined			12	14	90.6
Wang 2021	China	9977	2018	1046	61.2		Not defined				14.7	
Wilf Miron 2019	Israel	35,776	2015	4832	59.7		Not defined				8.5	
Willaing 2003	Denmark	33,441	1999	40			Not defined			23	25	
Wilson 2020	Australia			251			Family medicine				21.1	
Wyshak 1980	USA	11,674	1979	289	92		Not defined				13.8	
Yaacob 1993	Malaysia	2654	1991	120	70.8		Not defined			13.3	17.5	100
Yan 2008	China	1289	2003	358			Not defined			10.6	35.8	
Young 1997	Australia	21,861	1996	855			Family medicine				3.2	
Zabadi 2018	Paslestine		2005	502	80.1	34.92	Family medicine, Study, Others			12.2	39.6	64.8
Zanetti 1998	Italy	23,020	1996	393	74		Not defined				31	68.9
Zhang 2012	China	4550	2010	84	38.1	39.4	Not defined	17.6	82.4	2	20.2	
Zhang 2015	China	8069		8725			Study, Others				12.8	96.7
Zhou 2010	China	2694	2007	673	73.3		Not defined			5	26.2	96.6
Zinonos 2016	Cyprus	35,391	2008	119	59.7		Not defined			16	28.6	
Zylbersztejn 2015	Argentina	13,080	2013	3033		41.3	Not defined			21.7	19.7	

## Data Availability

All relevant data are within the paper.

## Supplementary Materials

The following are available online at https://www.mdpi.com/article/10.3390/ijerph182413328/s1: Figure S1, Funnel plots; Figure S2, Meta-analysis on prevalence of ex-smokers among physicians; Figure S3, Comparisons.

## Appendix A. Details for the Search Strategy Used within Each Database

**Pubmed**

“Smoking”[Mesh:NoExp] OR “smokings”[TW] OR “smoking”[TW] OR “Tobacco Smoking”[MH] OR “Smokers”[MH] OR “smoker”[TW] OR “Smokers”[TW] OR “tobacco”[Title]

AND

“family doctor “[TW] OR “general practitioner”[TW] OR “general practitioners”[TW] OR “practitioner, general”[TW] OR “practitioners, general”[TW] OR “general physician”[TW] OR “general physicians”[TW] OR “physicians, family”[TW] OR “family physicians”[TW] OR “family physician”[TW] OR “physician, family”[TW] OR “physicians, primary care”[TW] OR “physician, primary care”[TW] OR “primary care practitioner “[TW] OR “primary care physician”[TW] OR “primary care physicians”[TW] OR “medical practitioner”[TW] OR “physicians”[MH] OR “physicians”[Title] OR “physician”[Title] OR “doctor”[title] OR “doctors”[title] OR “practitioner” [Title] OR “practitioners”[Title]

AND

“prevalence”[MH] OR “prevalence”[TW] OR “prevalences”[TW] OR “epidemiology”[MH] OR “episode of care”[TW] OR “epidemiological”[TW] OR “epidemiologic”[TW] OR “epidemiology”[TW] OR “epidemiology”[SH] OR “frequency”[TIAB] OR “morbidity”[TIAB] OR “epidemics”[TIAB] OR “outbreaks”[TIAB] OR “endemics”[TIAB] OR “occurrence”[TIAB] OR “surveillance”[TIAB] OR “incidence”[TIAB]

**Embase**

‘health personnel attitude’/exp AND ‘smoking/exp AND ‘physician’/exp

**Cochrane**

“smokings”:ti,ab,kw OR “smoking”:ti,ab,kw OR “smoker”:ti,ab,kw OR “Smokers”:ti,ab,kw OR tobacco:ti,ab,kw

AND

“physicians, family”:ti,ab,kw OR “physicians, primary care”:ti,ab,kw OR “family doctor”:ti,ab,kw OR “general practitioner”:ti,ab,kw OR “general practitioners”:ti,ab,kw OR “practitioner, general”:ti,ab,kw OR “practitioners, general”:ti,ab,kw OR “general physician”:ti,ab,kw OR “general physicians”:ti,ab,kw OR “physicians, family”:ti,ab,kw OR “family physicians”:ti,ab,kw OR “family physician”:ti,ab,kw OR “physician, family”:ti,ab,kw OR “physicians, primary care”:ti,ab,kw OR “physician, primary care”:ti,ab,kw OR “primary care practitioner “:ti,ab,kw OR “primary care physician”:ti,ab,kw OR “primary care physicians”:ti,ab,kw OR “physicians”:ti,ab,kw OR “doctor”:ti,ab,kw OR “doctors”:ti,ab,kw OR “medical practitioner”ti,ab,kw OR “physicians”:ti,ab,kw OR “physicians”:ti OR “physician”:ti OR “doctor”:ti OR “doctors”:ti OR “practitioners”:ti OR “practitioner”:ti

AND

“prevalence”:ti,ab,kw OR “prevalences”:ti,ab,kw OR “epidemiology”:ti,ab,kw OR “episode of care”:ti,ab,kw OR “epidemiological”:ti,ab,kw OR “epidemiologic”:ti,ab,kw OR “frequency”:ti,ab,kw OR “morbidity”:ti,ab,kw OR “epidemics”:ti,ab,kw OR “outbreaks”:ti,ab,kw OR “endemics”:ti,ab,kw OR “occurrence”:ti,ab,kw OR “surveillance”:ti,ab,kw

**Table A1:** Methodological assessment of studies using STROBE and CONSORT criteria.

**STROBE**						
		**Total**	**Abstract**	**Methods**	**Results**	**Discussion**
		**Score**	**Introduction**			
Aaro 1977		60	100	36	60	75
Abdullah 2006		59	100	69	30	75
Aboyans 2009		56	100	31	50	100
Akvardar 2004		53	100	38	40	75
Al Alwan 2013		50	100	31	40	100
Alarjan 2015		53	100	46	40	75
al-Khateeb 1990		38	100	31	30	25
Allan 1976		13	0	NA	20	NA
Al-Lawati 2009		44	100	23	20	100
Al Shahrani 2021		69	100	69	60	75
An 2004		66	100	69	50	75
Amara 2008		53	100	38	40	100
Amte 2015		56	100	38	50	100
Arnetz 1988		66	100	54	60	75
Aryayev 2014		48	100	31	33	100
Baltaci 2014		53	100	38	40	75
Baptista 1993		44	100	31	30	100
Barengo 2004		45	100	42	33	50
Barengo 2005		44	100	23	30	100
Barnoya 2002		28	50	0	20	100
Basnyat 2000		34	100	10	20	100
Basu 2011		47	100	23	40	100
Behbehani 2004		50	100	54	20	75
Belkić 2007		59	100	38	50	100
Belkić 2012		56	100	54	30	100
Bener 1993		38	100	15	30	50
Borgan 2014		47	100	31	20	100
Bortz 1992		29	75	10	30	50
Bostan 2015		56	100	46	40	75
Bourke 1972		41	100	23	30	50
Braun 2004		41	100	31	30	25
Brenner 1996		56	100	46	40	100
Brink 1994		53	100	46	50	50
Brotonsc 2005		53	100	46	30	75
Burgess 1970		19	50	10	20	25
Burgess 1978		28	75	23	20	50
Cao 2011		41	50	25	44	75
Carlos 2020		70	100	62	60	75
Ceraso 2009		59	100	46	40	100
Chaudhry 2009		32	100	17	20	50
Cheng 1990		45	100	25	40	75
Coe 1971		34	50	15	30	75
Cofta 2008		50	100	38	30	75
Das 2013		53	100	46	30	75
Davies 1989		47	75	45	40	50
De Col 2010		59	100	54	50	75
Dekker 1993		44	100	31	40	50
De Oliveira 2013		56	100	46	40	100
Desalu 2009		47	100	46	30	50
Djalalinia 2011		47	100	46	20	75
Dodds 1979		38	50	38	43	50
Doll 1954		41	100	31	20	75
Doll 1964		44	100	38	30	50
Doll 1994		50	100	31	50	75
Doll 2004		53	100	31	50	75
Easton 2001		78	100	77	50	100
Easton 2001		61	100	54	56	75
Edwards 2008		63	75	46	70	75
Edwards 2018		66	75	46	70	100
Fadhil 2007		63	100	55	50	75
Fanello 1990		56	100	54	50	50
Fathi 2016		56	100	46	30	100
Fowler 1989		69	100	77	50	75
Franceschi 1986		39	50	31	50	25
Frank 1998		75	100	77	60	100
Frank 2009		63	75	54	50	100
Freour 2011		50	100	38	30	100
Garfinkel 1976		27	50	23	20	25
Grossman 1999		56	100	46	40	75
Gunes 2005		60	100	46	50	100
Gupta 2013		50	100	46	30	75
Hallett 1983		28	75	10	30	50
Hamadeh 1999		53	100	38	40	75
Han Zao Li 2008		56	100	46	40	100
Hay 1976		41	100	15	40	75
Hay 1998		53	75	31	60	75
Heloma 1998		56	100	46	40	75
Hensrud 1993		53	100	38	50	75
Hepburn 2000		59	100	62	40	75
Heponiemi 2008		59	100	46	40	100
Hidalgo 2016		56	100	46	40	75
Hill 1997		50	100	54	30	50
Hodgetts 2004		56	100	38	50	100
Hoseainrezae 2013		44	100	38	30	50
Huang 2013		63	100	46	50	100
Hughes 1991		63	100	64	40	75
Hughes 1992		63	75	69	40	75
Hughes 1999		50	100	46	20	100
Hung 2013		59	100	46	50	100
Hussain 1993		41	100	38	30	25
Içli 1992		34	100	31	20	25
Innos 2002		52	100	38	44	75
Jacot Sadowski 2009		73	100	82	50	75
Jiang 2007		63	100	69	50	50
Jiménez-Ruiz 2015		56	100	38	40	100
Jingi 2015		47	100	38	30	75
John 2003		41	100	31	10	75
Joossens 1987		38	75	31	40	25
Josseran 2000		47	100	46	10	100
Josseran 2005		59	100	46	40	100
Julião 2013		53	100	38	40	100
Kaetsu 2002		63	100	45	70	75
Kaetsu 2002		78	100	77	60	75
Kai 2008		59	100	69	30	75
Kaneita 2010		78	100	77	60	100
Kawahara 2000		70	100	64	50	100
Kawakami 1997		48	75	45	22	75
Kawane 1993		50	0	NA	NA	NA
Kono 1995		78	100	85	60	100
Kotz 2007		78	100	77	60	100
Lam 2011		88	100	92	70	100
La Vecchia 2000		20	100	15	20	NA
Lefcoe 1970		52	100	55	22	75
Legnini 1987		50	100	46	30	75
Lindfors 2009		66	100	69	40	100
Linn 1986		56	100	54	50	50
Lipp 1972		44	100	31	20	100
Lipp 1972		31	75	15	20	75
Magee 2017		63	100	62	30	100
Malik 2010		47	100	46	30	50
Manson 2000		63	100	62	40	100
Mappin-Kasirer 2020		63	100	62	50	75
Marakoğlu 2006		47	100	38	30	75
Márk 1998		53	100	46	30	100
Mathavan 2009		63	100	62	40	75
McAuliffe 1984		41	100	31	30	50
McEwen 2001		56	100	46	50	75
McGrady 2007		63	100	46	50	100
Mejia 2011		54	100	50	33	75
Merrill 2006		56	100	62	20	100
Meshefedjian 2010		59	100	54	40	100
Mikalauskas 2012		63	100	54	50	100
Misra 2004		63	100	62	50	75
Miwa 1995		50	100	46	30	75
Mohan 2006		50	100	46	40	50
Mohseni-Bandpei 2011		63	100	62	30	100
Moreno 2006		24	100	15	30	25
Mostafa 2017		63	100	62	40	100
Movsisyan 2019		56	100	62	40	50
Mubeen 2008		59	100	46	40	100
Naji 2006		34	100	15	30	50
Nakládalová 2005		50	100	38	40	50
Nardini 1998		53	100	54	30	50
Nawaz 2007		56	100	62	40	50
Nawaz 2008		44	100	31	30	50
Ndiaye 2001		56	100	54	50	50
Nelson 1994		65	75	77	22	100
Ng 2007		61	100	62	56	50
Nollen 2004		53	100	54	30	75
Nutbeam 1990		53	100	46	40	50
Obeidat 2017		66	100	54	50	100
Öztürk 2012		59	100	69	50	25
O’Cathail 2013		63	100	62	40	100
Ohida 2001		63	100	54	40	100
O’Keeffe 2019		75	100	69	60	100
Pärna 2005 A		69	100	69	40	100
Pärna 2005 B		66	100	62	40	100
Pärna 2017		59	100	46	40	100
Perrin 2006		63	100	62	40	100
Peykari 2010		59	100	46	40	100
Phillips 1968		41	100	31	30	50
Pillay 2020		47	50	31	50	50
Pipe 2009		59	100	54	30	100
Piryani 2004		34	100	15	30	50
Pizzo 2003		47	100	42	30	50
Poanta 2006		73	75	77	50	100
Põld 2017		72	100	69	50	100
Põld 2020		94	100	92	90	100
Polyzos 1995		38	50	38	40	25
Power 1999		66	100	77	40	75
Ramachandran 2008		70	75	73	50	75
Ranchal 2018		74	100	77	56	100
Rankin 1975		44	100	8	50	75
Ravara 2014		55	100	31	60	75
Reile 2018		74	100	46	58	100
Roche 1995		66	100	62	60	75
Roche 1996		69	100	77	60	50
Rurik 2008		47	100	31	40	75
Rurik 2014		41	100	31	30	50
Sachs 1983		44	50	67	44	0
Saeed 1991		53	75	38	50	75
Saeys 2014		81	100	77	70	100
Salgado 2014		72	75	69	80	100
Samuels 1997		50	75	23	50	100
Schnoll 2006		59	100	62	50	50
Scott 1992		41	100	23	30	75
Sebo 2007		65	100	62	44	100
Seller 1983		42	50	44	50	25
Senior 1982		56	100	62	30	75
Sharma 1988		24	50	15	17	100
Shkedy 2013		59	100	36	44	100
Shi 2010		61	100	67	30	100
Shin 2012		72	100	77	50	100
Shishani 2008		75	100	85	40	100
Shishani 2011		75	75	85	60	75
Siddiqui 2001		50	100	46	30	75
Singh 1981		50	100	38	30	75
Smith 2006		67	100	64	50	75
Smith 2007		50	67	50	33	NA
Sotiropoulos 2007		70	100	55	80	75
Squier 2006		63	100	54	50	100
Steinberg 2007		50	100	46	30	75
Stuyt 2009		55	75	31	56	100
Sundquist 1999		72	100	69	50	100
Svärdsudd 2002		69	100	77	50	75
Tapia-Conyer 1997		66	100	62	50	75
Tee 2007		69	100	62	60	75
Tessier 1996		53	100	38	30	100
Thankappan 2008		56	100	62	40	50
Thomas 1986		66	100	54	50	100
Thomas 1997		44	100	38	30	50
Tomson 2003		56	100	46	30	100
Tong 2010		66	100	62	40	100
Torre 2005		53	100	38	30	100
Tosun 2016		53	100	38	30	100
Trédaniel 1993		50	100	54	30	50
Ulbricht 2009		66	100	54	50	100
Unal 2017		63	100	62	40	75
Underner 2004		63	50	62	50	100
Underner 2006		63	75	62	50	75
Uysal 2007		69	75	77	60	75
Vanderhoek 2013		81	100	77	70	100
Vanphanom 2011		73	100	85	25	100
Varona 2005		42	100	30	57	25
Viegas 2007		63	75	69	38	75
Voigt 2009		63	75	62	56	75
Waalkens 1992		57	75	42	56	75
Wada 2007		66	100	77	40	50
Wada 2011		50	100	38	40	75
Wang 2021		59	75	69	50	50
Wilf Miron 2019		59	100	54	50	50
Waillaing 2003		77	100	92	50	100
Wilson 2020		69	100	69	60	75
Wyshak 1980		34	50	38	22	25
Yaacob 1993		69	100	62	50	100
Yan 2008		87	100	100	67	75
Young 1997		56	50	NA	50	NA
Zabadi 2018		81	100	85	56	100
Zanetti 1998		47	75	54	30	50
Zhang 2012		56	100	69	40	25
Zhang 2015		70	67	NA	67	NA
Zhou 2010		35	75	50	60	100
Zinonos 2016		100	100	100	100	100
Zylbersztejn 2015		78	100	85	50	100
**CONSORT**						
	**Total**	**Abstract**	**Methods**	**Results**	**Discussion**	**Other**
	**Score**	**Introduction**				
Glavas 2003	73	100	59	70	67	33
Saadat 2012	76	100	59	80	100	33

**Table A2:** Methodological assessment of studies using NOS criteria. NOS for cross-sectional studies.

	Selection Bias				Comparability Bias	Outcome Bias	

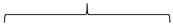
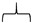
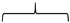
			
	Sample representativeness	Sample size	Representativeness	Ascertainment of the exposure	Comparability	Assessment of the outcome	Statistical test
Aaro 1977							
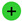
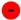
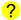
Abdullah 2006							
Aboyans 2009							
Akvardar 2004							
Al Alwan 2013							
Alarjan 2015							
al-Khateeb 1990							
Allan 1976							
Al-Lawati 2009							
Al Shahrani 2021							
Amara 2008							
Amte 2015							
An 2004							
Arnetz 1988							
Aryayev 2014							
Baltaci 2014							
Baptista 1993							
Barengo 2004							
Barengo 2005							
Barnoya 2002							
Basnyat 2000							
Basu 2011							
Behbehani 2004							
Belkić 2007							
Belkić 2012							
Bener 1993							
Borgan 2014							
Bortz 1992							
Bostan 2015							
Bourke 1972							
Braun 2004							
Brenner 1996							
Brink 1994							
Brotonsc 2005							
Burgess 1970							
Burgess 1978							
Carlos 2020							
Ceraso 2009							
Chaudhry 2009							
Cheng 1990							
Coe 1971							
Cofta 2008							
Das 2013							
Davies 1989							
De Col 2010							
Dekker 1993							
De Oliveira 2013							
Desalu 2009							
Djalalinia 2011							
Dodds 1979							
Doll 1954							
Doll 1964							
Doll 1994							
Doll 2004							
Easton 2001							
Easton 2001							
Edwards 2008							
Edwards 2018							
Fadhil 2007							
Fanello 1990							
Fathi 2016							
Fowler 1989							
Franceschi 1986							
Frank 1998							
Frank 2009							
Freour 2011							
Garfinkel 1976							
Grossman 1999							
Gunes 2005							
Gupta 2013							
Hallett 1983							
Hamadeh 1999							
Han Zao Li 2008							
Hay 1976							
Hay 1998							
Heloma 1998							
Hensrud 1993							
Hepburn 2000							
Heponiemi 2008							
Hidalgo 2016							
Hill 1997							
Hodgetts 2004							
Hoseainrezae 2013							
Huang 2013							
Hughes 1991							
Hughes 1992							
Hughes 1999							
Hung 2013							
Hussain 1993							
Içli 1992							
Jacot Sadowski 2009							
Jiang 2007							
Jiménez-Ruiz 2015							
Jingi 2015							
John 2003							
Joossens 1987							
Josseran 2000							
Josseran 2005							
Julião 2013							
Kaetsu 2002							
Kai 2008							
Kaneita 2010							
Kawahara 2000							
Kawakami 1997							
Kawane 1993							
Kotz 2007							
Lam 2011							
La Vecchia 2000							
Lefcoe 1970							
Legnini 1987							
Lindfors 2009							
Linn 1986							
Lipp 1972							
Lipp 1972							
Magee 2017							
Malik 2010							
Marakoğlu 2006							
Márk 1998							
Mathavan 2009							
McAuliffe 1984							
McEwen							
McGrady 2007							
Mejia 2011							
Merrill 2006							
Meshefedjian 2010							
Mikalauskas 2012							
Misra 2004							
Miwa 1995							
Mohan 2006							
Mohseni-Bandpei 2011							
Moreno 2006							
Mostafa 2017							
Movsisyan 2019							
Mubeen 2008							
Naji 2006							
Nakládalová 2005							
Nardini 1998							
Nawaz 2007							
Nawaz 2008							
Ndiaye 2001							
Nelson 1994							
Ng 2007							
Nollen 2004							
Nutbeam 1990							
Obeidat 2017							
Öztürk 2012							
O’Cathail 2013							
Ohida 2001							
O’Keeffe 2019							
Pärna 2005 A							
Pärna 2005 B							
Pärna 2017							
Perrin 2006							
Peykari 2010							
Phillips 1968							
Pillay 2020							
Pipe 2009							
Piryani 2004							
Pizzo 2003							
Poanta 2006							
Põld 2017							
Põld 2020							
Polyzos 1995							
Power 1999							
Ramachandran 2008							
Ranchal 2018							
Rankin 1975							
Ravara 2014							
Reile 2018							
Roche 1995							
Roche 1996							
Sachs 1983							
Saeed 1991							
Saeys 2014							
Salgado 2014							
Samuels 1997							
Schnoll 2006							
Scott 1992							
Sebo 2007							
Seiler 1983							
Senior 1982							
Sharma 1988							
Shkedy 2013							
Shi 2010							
Shin 2012							
Shishani 2008							
Shishani 2011							
Siddiqui 2001							
Singh 1981							
Smith 2006							
Smith 2007							
Sotiropoulos 2007							
Squier 2006							
Steinberg 2007							
Stuyt 2009							
Sundquist 1999							
Tapia-Conyer 1997							
Tee 2007							
Tessier 1996							
Thankappan 2008							
Thomas 1986							
Tomson 2003							
Tong 2010							
Tosun 2016							
Trédaniel 1993							
Ulbricht 2009							
Underner 2004							
Underner 2006							
Uysal 2007							
Vanderhoek 2013							
Vanphanom 2011							
Varona 2005							
Viegas 2007							
Voigt 2009							
Waalkens 1992							
Wada 2007							
Wada 2011							
Wang 2021							
Wilf Miron 2019							
Willaing 2003							
Wilson 2020							
Wyshak 1980							
Yaacob 1993							
Yan 2008							
Young 1997							
Zabadi 2018							
Zanetti 1998							
Zhang 2012							
Zhang 2015							
Zhou 2010							
Zinonos 2016							
Zylbersztejn 2015							

## Appendix B

**Table A3:** NOS for cohort studies.

	Selection Bias				Comparability Bias	Outcome Bias		
			
	Representativeness of the exposed	Selection of the non-exposed	Ascertainment of the exposure	Demonstration that outcome of interest was not present at start of study	Comparability	Assessment of the outcome	Follow-up long	Adequacy of follow-up
Cao 2011								
Innos 2002								
Kaetsu 2002								
Kono 1985								
Manson 2000								
Mappin-Kasirer 2020								
Rurik 2008								
Rurik 2014								
Svärdsudd 2002								
Thomas 1997								
Torre 2005								
Unal 2017								
