# Supplementary material for: Smoking Prevalence among Physicians: A Systematic Review and Meta-Analysis
Source: Int J Environ Res Public Health. 2021 Dec 17;18(24):13328. doi: 10.3390/ijerph182413328 (PMC8705497; doi:10.3390/ijerph182413328)
Supplement: Supplementary file 1 [file ijerph-18-13328-s001.zip › ijerph-1502223-supplementary.pdf]

Supplementary Materials:

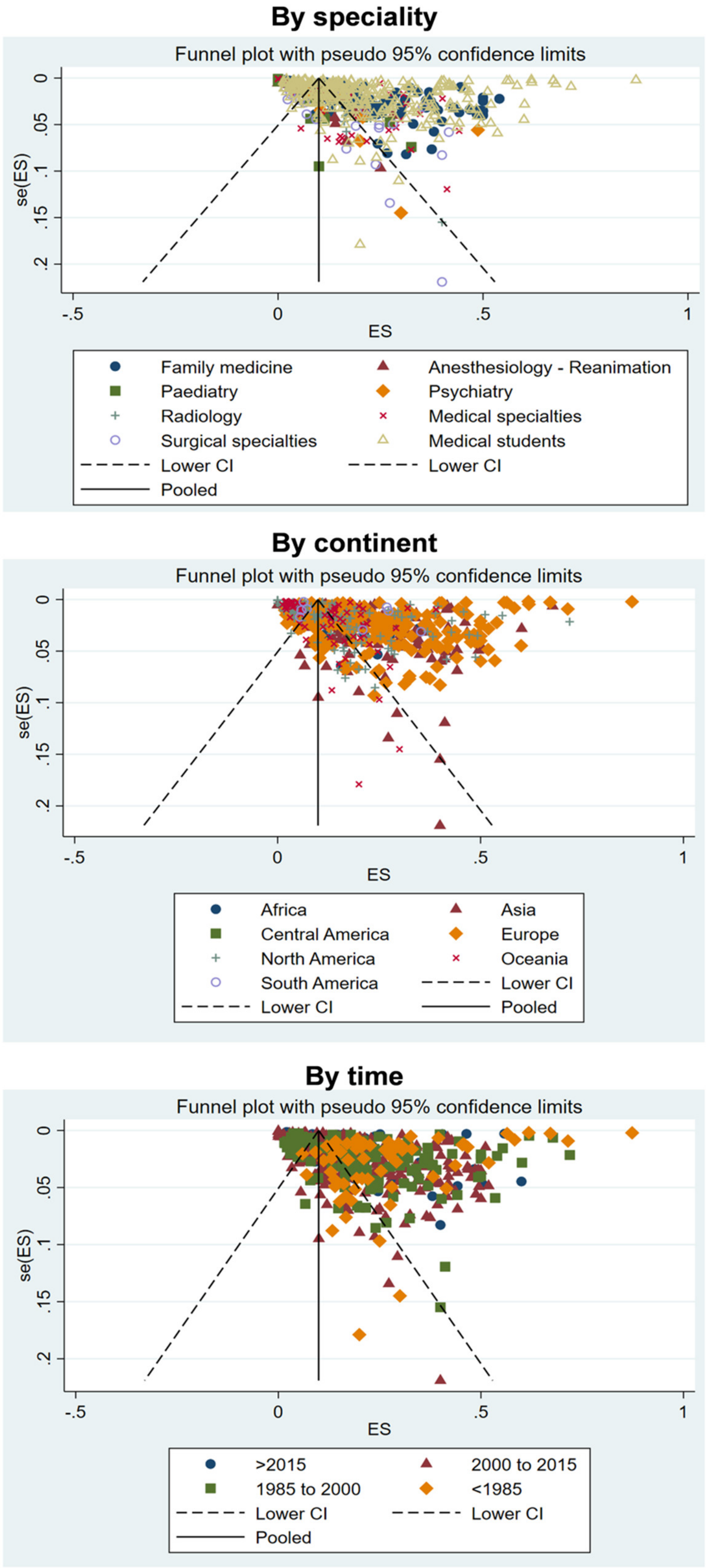

Figure S1. Funnel plots.

Variables	n studies (n subgroups)	Percentage of ex-smokers (95CI)		Weight (%)
**By speciality**				
**Medical students**	14 (15)	11%	(6 to 17%)	6,5
**Family medicine**	29 (31)	25%	(20 to 30%)	13,4
**Surgical specialities**				
Chest surgery	1 (1)	39%	(35 to 43%)	0,4
Ear Nose Throat	2 (2)	31%	(15 to 47%)	0,6
Gynecology	5 (5)	22%	(17 to 27%)	1,9
Orthopaedy	1 (1)	37%	(24 to 52%)	0,4
Overall surgical specialities	9 (9)	28%	(20 to 35%)	3,3
**Psychiatry**	3 (3)	29%	(19 to 40%)	1,2
**Medical specialities**				
Biology	1 (1)	20%	(14 to 29%)	0,4
Cardiology	3 (3)	35%	(22 to 48%)	1.3
Emergency	1 (1)	18%	(15 to 22%)	0,4
Internal medicine	7 (7)	24%	(16 to 32%)	2,9
Oncology	1 (1)	51%	(39 to 63%)	0,4
Pneumology	4 (4)	25%	(12 to 38%)	1,7
Public health	2 (2)	20%	(10 to 29%)	0,7
Overall medical specialities	19 (19)	27%	(21 to 33%)	7,9
**Anesthesiology**	4 (4)	13%	(11 to 16%)	1,6
**Radiology**	4 (4)	23%	(14 to 32%)	1,6
**Paediatry**	3 (3)	20%	(8 to 31%)	1,1
**Undefined**	101 (146)	23%	(20 to 26%)	63,1
**By continent**				
Europe	58 (111)	25%	(21 to 29%)	47,4
Asia	40 (57)	17%	(14 to 20%)	23,6
North America	21 (32)	27%	(20 to 34%)	13,7
South America	6 (6)	26%	(14 to 38%)	2,6
Africa	4 (4)	8%	(6 to 10%)	1,7
Oceania	7 (25)	20%	(17 to 23%)	11,0
**By time**				
> 2015	13 (32)	21%	(14 to 28%)	14,1
2000-2015	77 (132)	22%	(19 to 24%)	55,9
1985-2000	34 (55)	24%	(18 to 30%)	23,0
< 1985	11 (16)	31%	(26 to 35%)	7,0
**Overall**	**135 (454)**	**23%**	**(21 to 25%)**	**100.0**

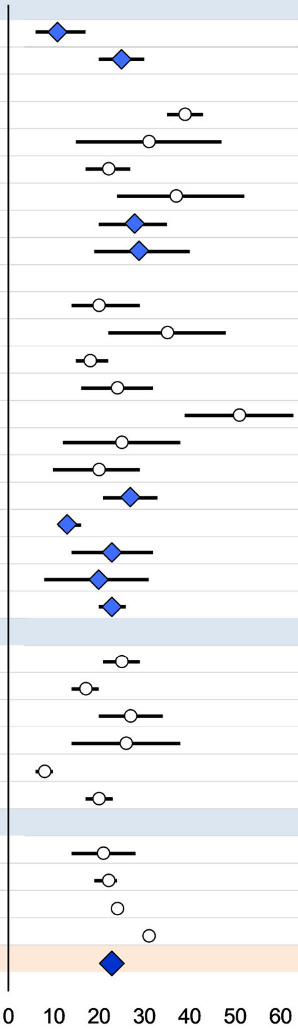

Figure S2. Meta-analysis on prevalence of ex-smokers among physicians.

	Coefficient (95% CI)	p-value
**Comparisons between speciality**		
Medical students vs Family medicine	-0,02 (-0.07 to 0.02)	0,312
" vs Surgical specialties	-0,05 (-0.15 to 0.04)	0,282
" vs Pscyhiatry	-0,07 (-0.21 to 0.08)	0,374
" vs Medical specialties	-0,04 (-0.10 to 0.02)	0,174
" vs Anesthesiology	-0,09 (-0.22 to 0.04)	0,186
" vs Radiology	-0,01 (-0.14 to 0.12)	0,858
" vs Paediatry	-0,03 (-0.19 to 0.13)	0,681
Family medicine vs Surgical specialties	-0,03 (-0.13 to 0.07)	0,600
" vs Pscyhiatry	-0,04 (-0.20 to 0.11)	0,585
" vs Medical specialties	-0,02 (-0.09 to 0.05)	0,631
" vs Anesthesiology	0,11 (-0.02 to 0.25)	0,105
" vs Radiology	0,01 (-0.13 to 0.15)	0,860
" vs Paediatry	0,06 (-0.11 to 0.22)	0,489
Surgical specialities vs Pscyhiatry	-0,02 (-0.19 to 0.16)	0,862
" vs Medical specialties	0,01 (-0.10 to 0.12)	0,862
" vs Anesthesiology	0,14 (-0.02 to 0.30)	0,084
" vs Radiology	0,04 (-0.12 to 0.20)	0,628
" vs Paediatry	0,08 (-0.10 to 0.27)	0,362
Pscyhiatry vs Medical specialties	0,02 (-0.13 to 0.18)	0,755
" vs Anesthesiology	0,15 (-0.04 to 0.35)	0,120
" vs Radiology	0,05 (-0.14 to 0.25)	0,584
" vs Paediatry	0,10 (-0.12 to 0.32)	0,361
Medical specialities vs Anesthesiology	0,13 (-0.01 to 0.27)	0,071
" vs Radiology	0,03 (-0.11 to 0.17)	0,681
" vs Paediatry	0,08 (-0.09 to 0.24)	0,379
Anesthesiology vs Radiology	-0,10 (-0.28 to 0.08)	0,286
" vs Paediatry	-0,05 (-0.26 to 0.15)	0,600
Radiology vs Paediatry	0,05 (-0.16 to 0.25)	0,664
**Comparisons between continent**		
Europe vs Asia	0,08 (0.04 to 0.12)	**<0.001**
" vs North America	-0,02 (-0.07 to 0.03)	0,460
" vs South America	-0,01 (-0.11 to 0.09)	0,830
" vs Africa	0,17 (0.05 to 0.29)	**0,007**
" vs Oceania	0,05 (0.00 to 0.10)	0,052
Asia vs North America	-0,10 (-0.15 to -0.05)	**<0.001**
" vs South America	-0,09 (-0.19 to 0.01)	0,069
" vs Africa	0,08 (-0.04 to 0.21)	0,174
" vs Oceania	-0,03 (-0.09 to 0.03)	0,279
North America vs South America	0,01 (-0.10 to 0.11)	0,890
" vs Africa	0,18 (0.06 to 0.31)	**0,004**
" vs Oceania	0,07 (0.01 to 0.13)	**0,031**
South America vs Africa	0,18 (0.03 to 0.31)	**0,022**
" vs Oceania	0,06 (-0.04 to 0.17)	0,250
Africa vs Oceania	-0,12 (-0.24 to 0.01)	0,073
**Comparisons between time**		
> 2015 vs 2000-2015	0,00 (-0.05 to 0.04)	0,880
" vs 1985-2000	-0,03 (-0.08 to 0.02)	0,278
" vs < 1985	-0,09 (-0.17 to -0.02)	**0,012**
2000-2015 vs 1985-2000	-0,03 (-0.07 to 0.01)	0,193
" vs < 1985	-0,09 (-0.16 to -0.03)	**0,005**
1985-2000 vs < 1985	-0,06 (-0.13 to 0.00)	0,066
**Sociodemographic**		
Age. years	-0,01 (-0.01 to -0.01)	**<0.001**
Gender. male as reference	-0,01 (-0.01 to 0.00)	**<0.001**

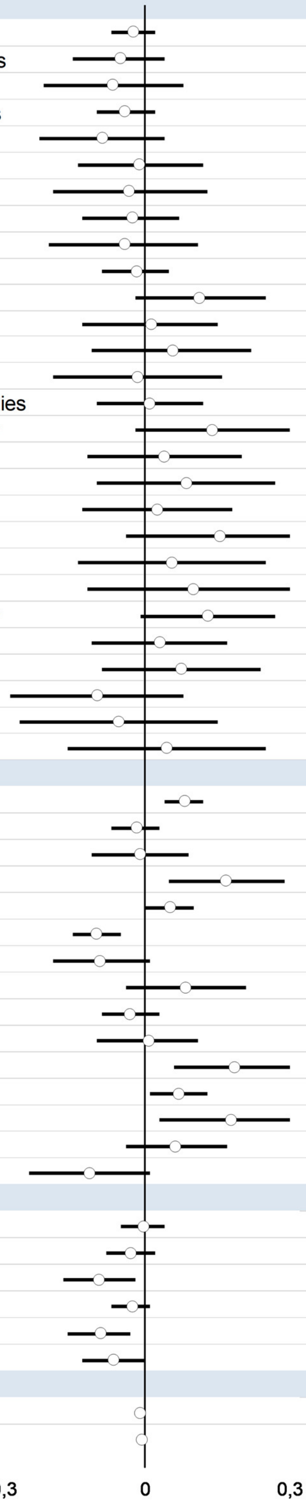

Figure S3. Comparisons.
